# Solvent-free electrically conductive Ag/ethylene vinyl acetate (EVA) composites for paper-based printable electronics†

**DOI:** 10.1039/c9ra02593f

**Published:** 2019-06-21

**Authors:** Yuqiu Shen, Zhenxing Chen, Yong Zhou, Zuomin Lei, Yi Liu, Wenchao Feng, Zhuo Zhang, Houfu Chen

**Affiliations:** School of Chemical Engineering and Technology, Sun Yat-sen University Tangjiawan Zhuhai 519082 P. R. China 2764927916@qq.com 3362104303@qq.com; The Key Laboratory of Low-Carbon Chemistry & Energy Conservation of Guangdong Province, Sun Yat-sen University Guangzhou 510275 P. R. China

## Abstract

Solvent-free electrically conductive composites have been applied to flexible electronics to obtain high electrical conductivity. However, some of the proposed composites have low electrical conductivities and are unable to meet the requirements of commercial printable electronics. In this study, solvent-free electrically conductive Ag/EVA (ethylene vinyl acetate) composites for paper-based printable electronics were prepared by a thermal melting method. The properties of these electrically conductive Ag/EVA composites, including particle sizes, morphologies and phase purities of the flake silver flake powders, were investigated using a particle size analyzer, scanning electron microscopy (SEM) and X-ray diffraction (XRD), respectively. The results showed that nanometer-thick flake silver flake powders with smooth and flat surfaces were made by the nanofilm transition technique. These obtained powders were able to form smooth face-to-face contacts, which facilitated the formation of an excellent conductive network in the conductive system. Dynamic mechanical analysis (DMA) was conducted to investigate the mechanical properties of EVA and Ag/EVA composites. A Fourier transformation infra-red (FTIR) spectrometer, laser micro-Raman spectrometer and thermogravimetric analyzer were used to analyze the organic functional groups, glass transition temperatures and thermal weight losses of the EVA resin and solvent-free electrically conductive composites. The solvent-free electrically conductive Ag/EVA composite, which contained 55 wt% of the as-prepared flake silver flake powders, was found to have an extremely low volume resistivity of 1.23 × 10^−4^ Ω cm as well as excellent bending performance and adhesion. These features indicate the great potential of these composites for application in printed electronics.

## Abstract 2

A novel type of solvent-free electrically conductive Ag/EVA composite for paper-based printable electronics was prepared through a thermal melting method.

## Graphical abstract

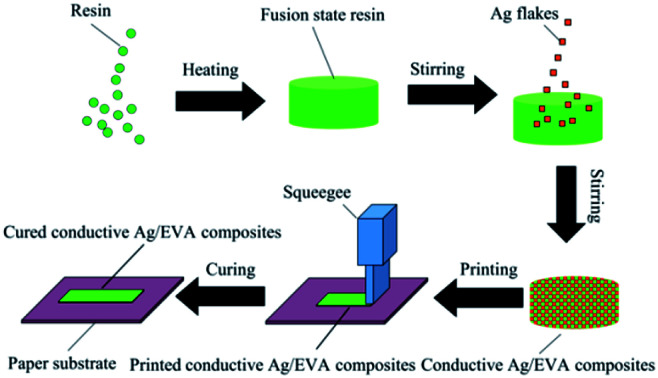

## 1. Introduction

Currently, electrically conductive composites filled with metallic or carbon particles have been increasingly applied in the field of printable electronics,^1,2^ for example, printed circuits, solar cells, radio frequency identification (RFID) tags, thin film transistors (TFT), sensor arrays, organic field effect transistors, microelectromechanical systems (MEMS), *etc.*^3–5^ With the rapid acceleration of industrialization, printed circuits with excellent performance characteristics,^6^ such as high electrical conductivity,^7^ lightweight, flexibility and superior mechanical stability,^8^ are required.

Meanwhile, with the rapid development of microelectronics, printable electronics on a variety of substrates, such as silicon,^9^ polyvinylidene fluoride (PVDF),^10^ polyethylene glycol terephthalate (PET),^11^ glass,^12^ paper,^13^ and polyester,^14^ have been achieved. As a potential substrate for flexible and printable electronics, paper has been intensively studied because of its merits, such as efficient cost, light weight, earth abundance, environmental benignity, foldability and biodegradability.^15,16^

Currently, numerous investigations on paper-based printed electronics have been carried out. Omniphobic fluoroalkylated paper was selected as the substrate for micro-electromechanical systems (MEMS) and electrodes, which were breathable, collapsible, and resistant to spoiling by exposure to water or other ordinary solvents.^17^ Paper-based multilayer printed circuit boards (P-PCBs) were fabricated using polyurethane (PU)-based electrically conductive adhesives (ECAs) through screen printing, and the life cycle assessment results showed that the P-PCBs had an approximately two order of magnitude lower effect on the environment than did O-PCBs (organic printed circuit boards).^18^ A multistep room temperature post-processing (MRTP) method was chosen for the fabrication of printed silver tracks (paper-based), of which the electrical conductivity was nearly 2 times that of printed silver tracks cured under a conventional heating process of 120 °C for 160 minutes.^19^

Typically, electrically conductive composites consist of conductive micro/nanoscale particles, a polymer binder and organic solvents.^20^ In some cases, a coupling agent, thickener or other additives may be added as well.^21^ As part of the composition of electrically conductive composites, organic solvents play a key role in dissolving and dispersing the solid resins. After printing, the organic solvents should be evaporated. Considering the vapors of volatile organic compounds (VOCs), which can be inflammable and explosive for volatile organic solvents, it is very important to prepare solvent-free electrically conductive composites. Electrically conductive composites have been studied for decades,^22^ and there are several reports regarding solvent-free types. A new kind of electrically conductive composite was fabricated by using silver-coated wollastonite fibers as a filler with EVA copolymers. Once the filler fraction reached 29 vol%, the volume resistivity was 5.56 × 10^−3^ Ω cm.^23^ New kinds of electrically conductive polymeric composites were prepared by the inclusion of silver-coated polyamide (PA) particles in a high-density polyethylene (HDPE) matrix. The volume resistivity of these composites reached a value of 1.47 × 10^−3^ Ω cm when filled with 32.9 vol% of the silver-coated polyamide.^24^ Electrically conductive polyethylene terephthalate (PET)/graphene composites were manufactured by melt compounding at 285 °C with a mixer, and the volume resistivity of the composites reached 47.3 Ω cm when the filling fraction was 3.0 vol%.^25^

From the above studies on solvent-free electrically conductive composites, it can be seen that the electrical conductivities of this class of electrically conductive composites needs to be improved. With the rapid progress of the electronics industry, printable electronics with high electrical conductivities are required (for commercial electronics, the standard for volume resistivity is 1.0 × 10^−4^ Ω cm). In this study, we designed solvent-free electrically conductive Ag/EVA composites, which could be printed onto normal paper through heating. The advantages of this method aim to achieve a cost-effective and eco-friendly solution to fabricating highly electrically conductive paper-based printable electronics.

## 2. Experimental section

### 2.1 Materials

Silver wires, which were the raw materials used for preparing the silver powders, were purchased from Shenzhen Ulster industrial materials co., LTD. The fabrication of the silver flake powders is shown in the next paragraph. Commercial silver flake powders (FAgL 6500 and FAgL 6501) were purchased from Sino-Platinum Metals Co., Ltd. Ethylene vinyl acetate (EVA) copolymer was selected as the binder resin (Aladdin), in which the vinyl acetate (VA) content is 32% and the melt flow rate (MFR) is 0.43 g min^−1^ (at 190 °C for 2.16 kg). Potassium bromide (spectrographic grade) was purchased from Aladdin. Ethanol (analytical reagent, 99.7%) was purchased from Adamas Reagent Co., Ltd. A4 printing paper was purchased from Deli Group.

### 2.2 Fabrication of silver flake powders

The as-prepared silver flake powders, which had smooth surfaces and identical nanothicknesses, were fabricated by nanofilm transition method. The detailed preparation process is as follows.

At first, water-soluble resin solution was coated on polyethylene terephthalate (PET) substrate though screen printing method. After the smooth resin layer was cured on PET substrate, the substrate with resin layer was fixed on the turnplate in the vacuum coating equipment. Then a certain amount of silver wire was placed into the tungsten boat under the turnplate. Once the specified vacuum was reached, the silver wire was melted and gasificated, silver vapour would be deposited on the layer of water soluble resin and the thickness of silver film could be controlled about 40–100 nm. The silver films were fallen off from PET substrate as coarse silver powders when immersing in the deionized water. The coarse powders were further mashed into fine powders using ultrasonic techniques with an ultrasonic power density of 5 W m^−2^. To distinguish the connection between particle size and conductivity, the ultrasonication times varied from 15–90 minutes. After ultrasonic treatment, the fine silver flakes were separated from solution *via* vacuum filtration.^26,27^

### 2.3 Preparation of solvent-free electrically conductive Ag/EVA composites

**Fig. 1:**
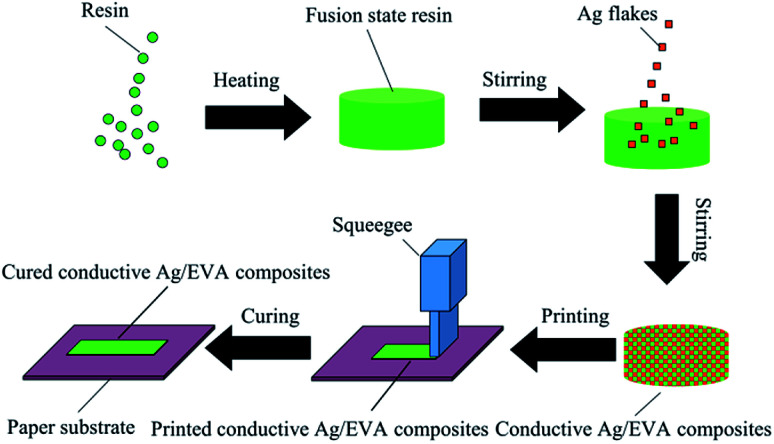
Manufacturing procedure for the solvent-free electrically conductive Ag/EVA composites.

A schematic of the fabrication of the solvent-free electrically conductive Ag/EVA composites is shown in Fig. 1. A quantitative amount of the solid resin was first weighed into a beaker and placed in a vacuum drying oven and then heated to 160 °C until all the solid resin was converted into a fused state. Corresponding amounts of the silver flake powders were subsequently added to the resin. Then, the container was removed from the vacuum drying oven and placed into an 80 °C water bath under normal atmosphere. Afterwards, the mixture was stirred at 160 rpm for 30 minutes, then a homogeneous conductive paste was formed (Fig. S1†), in which the filler loading of the silver flake powders in the conductive mixture was fixed to 40–70 wt%, depending on the sample. Then, the paste of the conductive Ag/EVA composites were used to print a pattern onto the substrate, which was plant fiber paper (detail information see the ESI†), by screen printing. After cooling down to room temperature, the printed pattern adhered to the paper substrate and possessed excellent electrical conductivity.

### 2.4 Electrical conductivity measurements

To calculate the volume resistivity of the conductive Ag/EVA films, we chose a 50 mm (length, *L*) × 10 mm (width, *w*) conductive pattern (Fig. S2†). After the volume resistance (*R*) and the thickness (*t*) of the samples were measured, the volume resistivity (*ρ*) could be calculated using the following formula:^28,29^
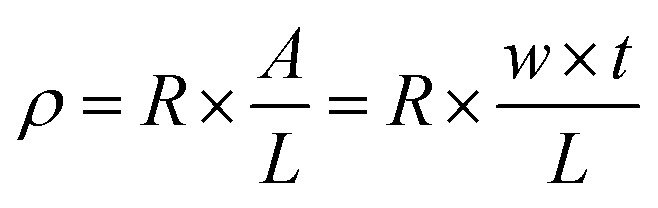
where *A* is the cross-sectional area of the conductive Ag/EVA composites, *R* is the volume resistance and *t* is the thickness.

### 2.5 Bending experiments

**Fig. 2:**
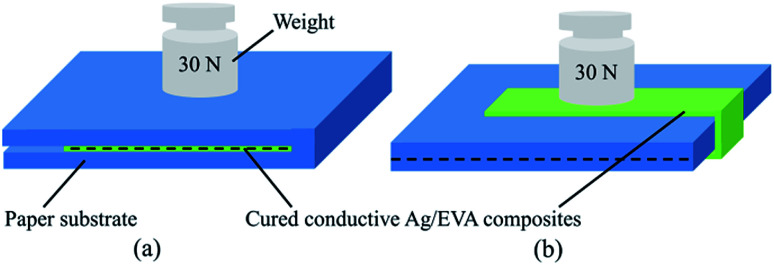
Images of the bending experiments for the conductive films folded (a) inside and (b) outside.

The bending performance of the solvent-free electrically conductive Ag/EVA composites was tested and is shown in Fig. 2. One bending cycle consisted of two folds (divided into inside and outside of the printed conductive films)^17,30^ under a pressure of 30 N for 60 seconds. Different places were chosen for the different cycles.

### 2.6 Adhesion test

We utilized an adhesion test in accordance with the international standard (ASTM D3359),^31,32^ in this standard, adhesion strength was rated according to a scale from 0B (weakest) to 5B (strongest). The conductive Ag/EVA composites film (as-prepared) was first crosshatched using a razor blade, then the adhesion of conductive Ag/EVA composites was assessed by inspecting the films after removal of the Scotch tape (3M).

### 2.7 Characterization

The morphologies of the silver flake powders and solvent-free electrically conductive Ag/EVA composites were analyzed by a field emission SEM (JSM-6330F, Japan). The particle sizes of the silver flake powders were measured with a Master Sizer 2000 (UK). The X-ray diffraction study of the silver powders was performed by a D-MAX 2200 VPC X-ray diffractometer (RIGAKU 670, Japan) using Cu Kα radiation and *λ* = 1.54 Å. The samples were scanned in the 2*θ* range from 10° to 80° with a scanning rate of 10° min^−1^. The chemical nature of the organic functional groups of the EVA resin and solvent-free electrically conductive Ag/EVA composites were characterized by infrared (IR) spectrometry (PerkinElmer, Spectrum Two). The samples along with potassium bromide powder were pressed into transparent wafers for the infrared test. The Fourier transformation infra-red (FTIR) spectra were recorded using a Equinox-55 FTIR spectrometer (Bruker, Germany) equipped with a attenuated total reflectance (ATR) attachment. The organic functional groups of paper substrate were observed and assigned with ATR-FTIR. Scanning was conducted from 4000 to 400 cm^−1^ at 2 cm^−1^ intervals. Raman spectra were obtained by using a laser micro-Raman spectrometer (Renishaw inVia) equipped with a 632.8 nm HeNe laser with a 1 cm^−1^ spectral resolution. The thermal weight loss of each sample in air was studied using a thermogravimetric analyzer (TG209F1 Libra) from NETZSCH Instruments. The temperature was raised from room temperature (28 °C) to 500 °C at a heating rate of 10 °C min^−1^. The glass transition temperatures (*T*_g_) of the samples were analyzed by differential scanning calorimetry (DSC-214, NETZSCH Instruments) with a heating rate of 10 °C min^−1^ in a nitrogen atmosphere. The dynamic mechanical properties were evaluated using a TA Q850 dynamic mechanical analyzer in single cantilever mode at a fixed frequency of 1 Hz. Measurements were carried out at heating rate of 5 °C min^−1^ with temperature range from −60 to 60 °C. The specimen dimensions were 4.0 mm thick, 14.0 mm wide and 17.5 mm long. The thicknesses of the samples were tested by a film thickness gauge (CH-1-ST, China) with an accuracy of 1 μm. The resistances of the samples were measured using a low DC resistance tester (TH2512B, China).

## 3. Results and discussion

### 3.1 Characterization of silver flake powders

**Fig. 3:**
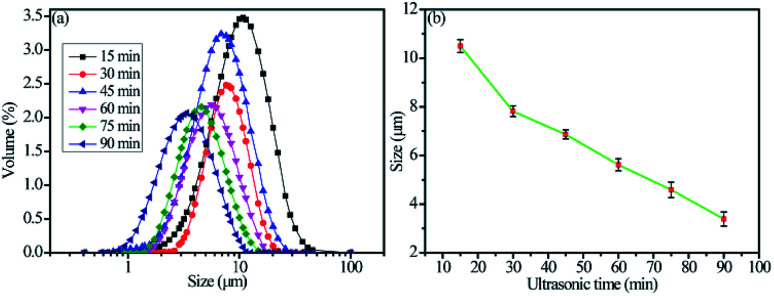
Particle sizes of the as-prepared flake silver powders: (a) particle size distributions and (b) mean particle sizes.

The particle sizes of the as-prepared flake silver powders were tested using a Mastersizer 2000 laser diffraction particle size analyzer, and the results are shown in Fig. 3. As the ultrasonication time was increased, the particle size gradually decreased. It can be seen in Fig. 3(a) that all of the silver flake powders treated at different ultrasonication times had narrow size distributions. After ultrasonication for 60 minutes, the average particle size of the silver flake powder decreased to 5.6 μm, which was suitable for the printable electronics.

**Fig. 4:**
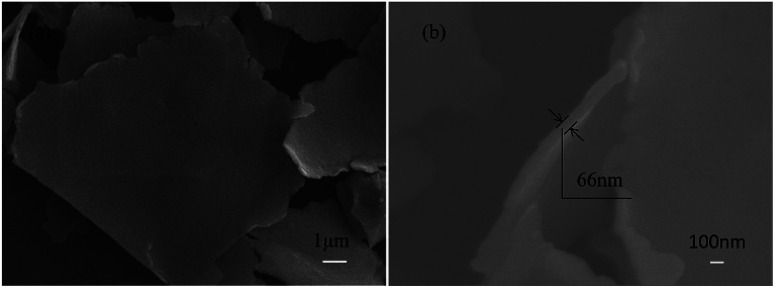
SEM images of the as-prepared silver flakes that were ultrasonically treated for 60 minutes: (a) surface appearance and (b) cross-section.

For the morphology of as-prepared silver flakes that were ultrasonically treated for 60 minutes (Fig. 4(a)), we found that the silver flake powder particles exhibited highly smooth and flat surfaces with fairly consistent thicknesses of approximately 66 nm (Fig. 4(b)).

**Fig. 5:**
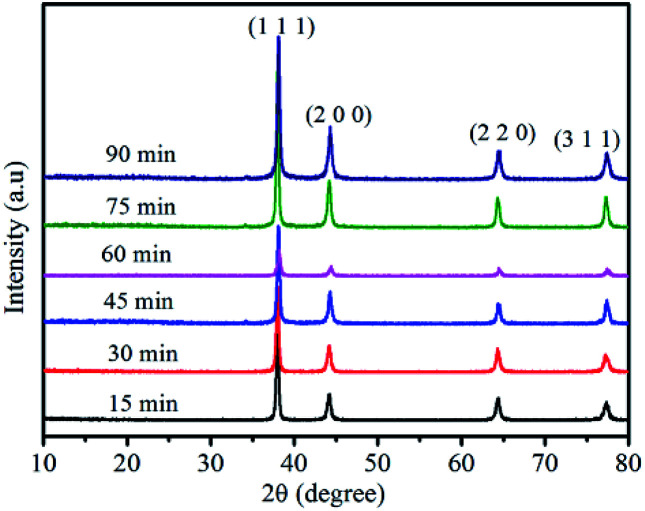
XRD of the as-prepared silver flakes after ultrasonic treatment for different lengths of time.

The silver powders that were ultrasonically treated for different lengths of time were further characterized using X-ray diffraction analysis (Fig. 5). All the prominent peaks were located at approximately 38.1°, 44.5°, 64.5° and 77.4°, respectively representing the (111), (200), (220) and (311) crystal facets of a silver unit cell with an fcc structure.^33,34^ Moreover, no silver oxide peaks were observed, which revealed that pure silver powders had been prepared by the nanofilm transition method.

### 3.2 The electrical conductivities and mechanical properties of the solvent-free electrically conductive Ag/EVA composites

**Fig. 6:**
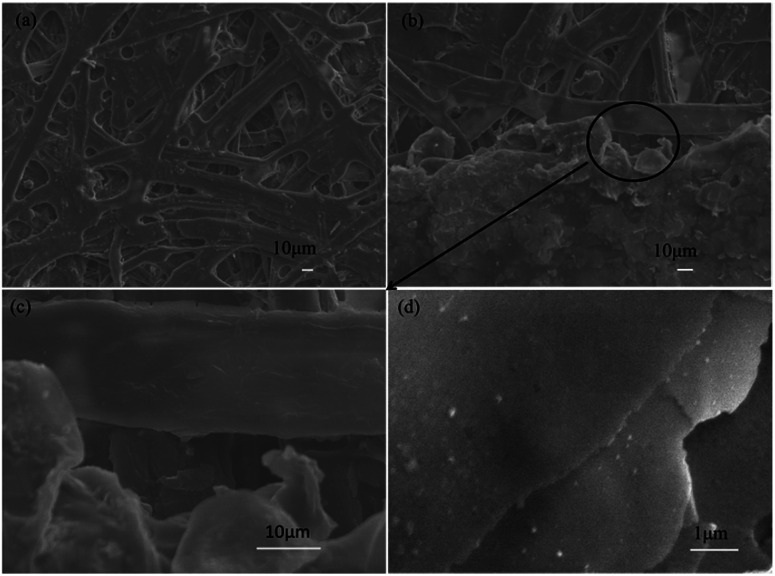
SEM images of the (a) paper substrate, (b) junction between the as-prepared conductive Ag/EVA composites and paper, (c) magnified border between the composites and substrate and (d) face-to-face conduction mode.

Because of its countless number of pores and numerous cellulose fibers (Fig. 6 and S5†), normal printing paper was chosen to be the substrate for printing the electrically conductive films. Once the melted electrically conductive Ag/EVA composites were printed onto the paper, the pastes could easily penetrate into the pores of the paper, resulting in strong adhesion (Fig. S6†).

**Fig. 7:**
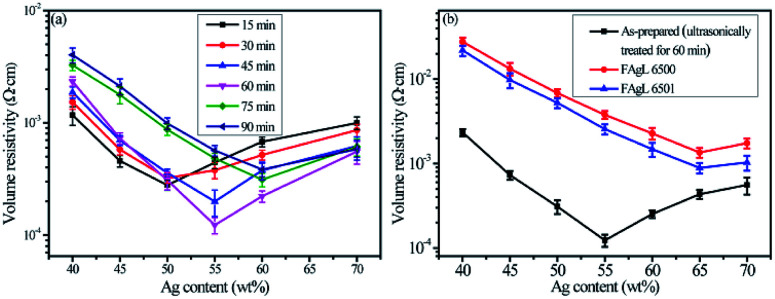
Volume resistivity as a function of Ag content in printed patterns of the solvent-free electrically conductive Ag/EVA composites (cured at room temperature). The conductive prints had fills of (a) as-prepared silver flake powders with different ultrasonic treatment times or (b) different types of silver flake powders.

The effects of Ag powder loading on the volume resistivity are shown in Fig. 7. As the silver powder loading was varied over the range of 55–60 wt%, the volume resistivity was relatively low. For the electrically conductive Ag/EVA composites, in which the silver flakes were ultrasonically treated for 60 minutes and the silver flake powder loading was 55 wt%, the volume resistivity reached 1.23 × 10^−4^ Ω cm. In contrast, FAgL 6500 and FAgL 6501, two commercial silver flake powders, showed volume resistivities of 1.35 × 10^−3^ Ω cm and 8.89 × 10^−4^ Ω cm when the silver powder loading was 65 wt%, respectively (Fig. 7(b)). The volume resistivity of the electrically conductive Ag/EVA composites fabricated from the prepared silver flakes was close to that of the conductive patterns printed using industrial silver powders with a 70 wt% filling rate for the silver flake powders.^35^ The excellent electrically conductive of our Ag/EVA composites was mainly attributed to the face-to-face contact pattern between the fine silver flakes, which had a uniform nanoscale thickness and smooth surfaces (Fig. 4(a)), that was beneficial to the further formation of an electrically conductive network (Fig. 6(d)).

**Fig. 8:**
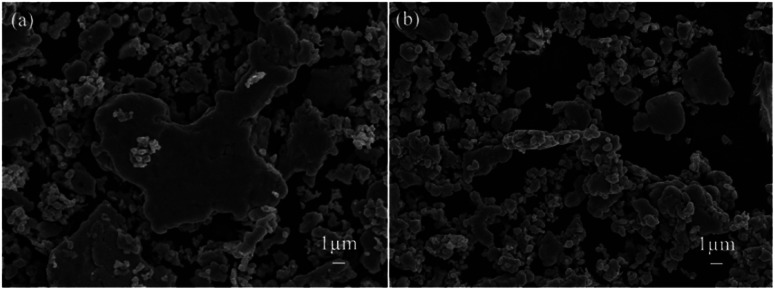
SEM images of commercial silver powders (a) FAgL 6500 and (b) FAgL 6501.

For FAgL 6500 and FAgL 6501, a number of agglomerates (Fig. 8(a)) with jagged edges (Fig. 8(b)) existed, which greatly influenced the dispersion of these silver powders. This led to a deterioration in the conductivity of their respective electrically conductive Ag/EVA composites.

In contrast, the prepared silver flake powders possessed smooth surfaces, identical nanoscale thicknesses and narrow particle size distributions, which in turn benefited the formation of ideal face-to-face contacts (Fig. 6(d)) that substantially improve the electrical conductivity. From Fig. 7(b), we can see that as the filling rate of the silver powders increased, the electrical conductivity deteriorated. Taking the as-prepared samples as an example, once the fill fraction of the prepared silver powders surpassed 55 wt%, the electrical conductivity worsened. Additionally, the viscosity of the pastes increased as the fill fraction of the silver powders exceeded 55 wt%, which resulted in an irregular arrangement of the silver powder throughout the conductive system (Fig. S3†). This decreased the effective contact area between the silver flakes and affected the internal conductive network, which led to the deterioration of the conductivity of the electrically conductive Ag/EVA composites.

Because these solvent-free electrically conductive composites based on the as-prepared silver flake powders have great potential for use in flexible electronics, it is important to investigate the conductive stability of the printed patterns in a bent state.

**Fig. 9:**
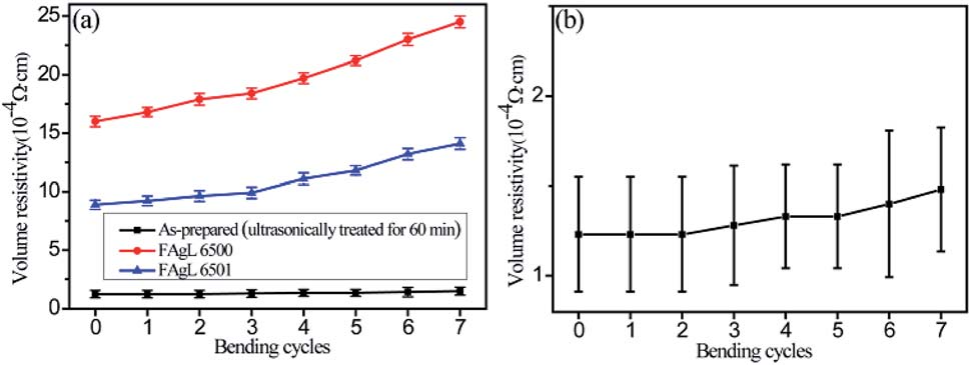
Images of the (a) volume resistivity of the solvent-free electrically conductive Ag/EVA composite as a function of the number of bending cycles and (b) magnification curve of the composites filled with the as-prepared silver flake powders.

The volume resistivity of the electrically conductive Ag/EVA composite prepared with the as-prepared silver flakes increased slightly after 7 cycles, which was a significantly lower than the rate of increment of conductive Ag/EVA composite filled with FAgL 6500 and 6501 (Fig. 9(a)).

**Fig. 10:**
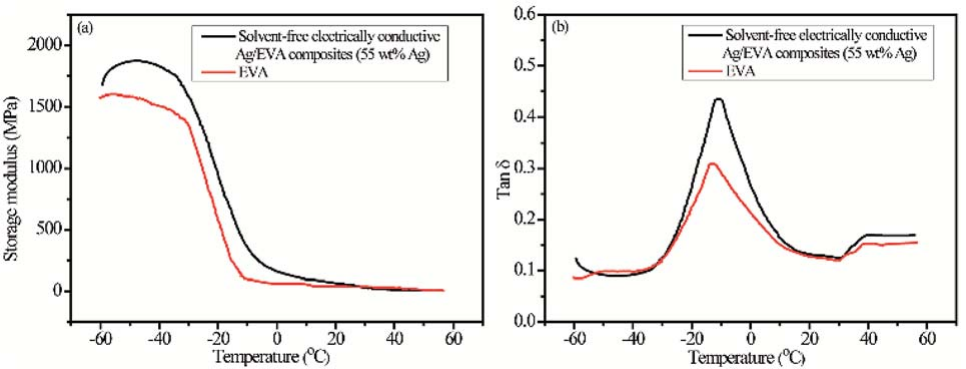
Dynamic mechanical analysis (DMA) of solvent-free electrically conductive Ag/EVA composites (filling with as-prepared silver powders) and EVA as a function of temperature (a) storage modulus and (b) damping factor (tan *δ*).

A further investigation was carried out on the dynamic mechanical of solvent-free electrically conductive Ag/EVA composites (55 wt% as-prepared Ag) and EVA. The stiffness of the material could be inferred from the value of the storage modulus (*E*′, elastic response). It can been seen from Fig. 10(a), the storage modulus of the samples deceased with the increasing of temperature, moreover, the storage modulus of Ag/EVA composites is slightly larger than EVA at the same temperature. The phenomenon indicating that the stiffness of Ag/EVA composites was enhanced slightly compared with EVA. The damping or loss factor (tan *δ*) could be determined by the ratio between the loss modulus *E*′′ (viscous response) and the storage modulus. The tan *δ* value is usually affected by loading, type and distribution of fillers, porosity of composites and filler–matrix interaction.^36^ The variations of the damping factor *versus* temperature for Ag/EVA composites and EVA are presented in Fig. 10(b). The incorporation of flaky silver powders into EVA matrix can increase the mechanical damping of the polymer. The increasing of tan *δ* may be ascribed to the restricted movement of the host polymer by interaction forces between additives (silver powders) and EVA. Higher tan *δ* of the Ag/EVA composites clearly indicates that it shows more viscous than elastic nature as compared with pure EVA. Generally, the temperature at maximum of tan *δ* is regarded as the glass transition temperature (*T*_g_).^37^ The tan *δ* peak of EVA exhibits a low temperature (−14 °C). The tan *δ* peak of Ag/EVA composites show a shifting to higher temperature about 3 °C.

### 3.3 Characterization of the EVA resin and solvent-free electrically conductive Ag/EVA composites

**Fig. 11:**
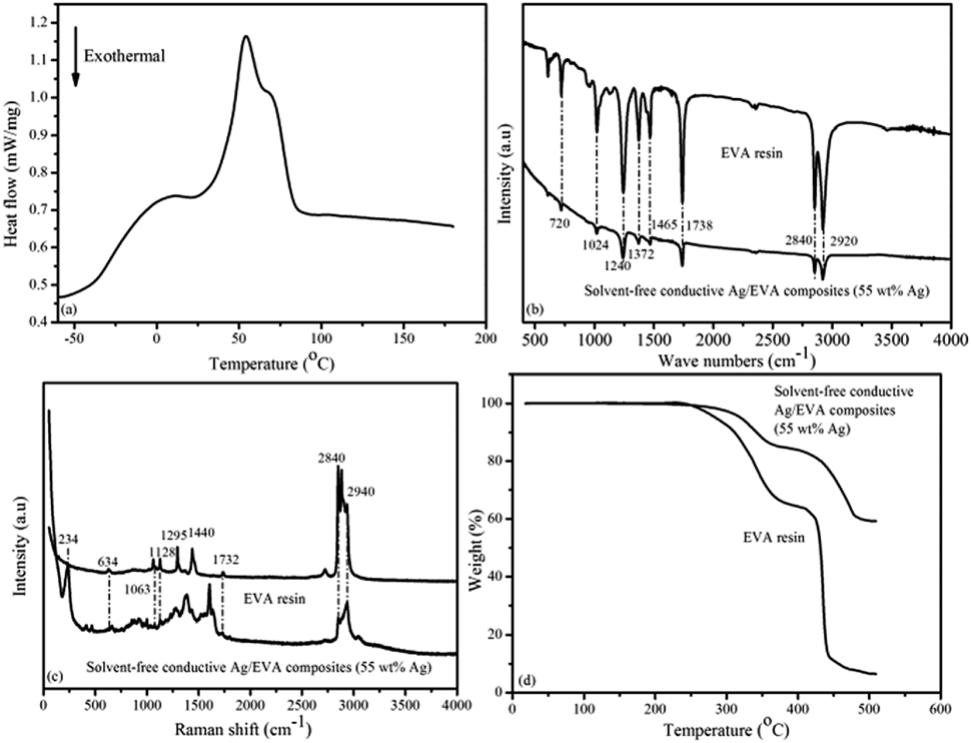
(a) DSC curve of the EVA resin during the heating cycle and (b) IR spectra, (c) Raman spectra and (d) TGA curves of the EVA resin and the solvent-free electrically conductive Ag/EVA composite (55 wt% Ag).

Differential scanning calorimetry (DSC) analysis of the EVA resin sample (Fig. 11(a)) suggests that the glass transition temperature (*T*_g_) of the EVA sample was approximately −21.4 °C, which is close to the results of previous research,^38,39^ and about 7.4 °C lower than the DMA result. The low *T*_g_ of EVA renders it an excellent flexibility at room temperature when these electrically conductive Ag/EVA composites are used as conductive films.

The IR spectra of the EVA resin and solvent-free electrically are shown in Fig. 11(b). The peaks at 2920 and 2840 cm^−1^ were attributed to antisymmetric and symmetric C–H stretching of the –CH_2_– groups.^40,41^ The peaks at 1738 cm^−1^ were ascribed to the stretching vibration of C=O in the ester groups (–COO–).^42^ The peaks at 1465 cm^−1^ were attributed to the in-plane deformation of the –CH_2_– groups.^43^ The presence of methyl groups was verified by the in-plan deformation peak at 1372 cm^−1^.^44^ The peaks at 1240 and 1024 cm^−1^ corresponded to the stretching vibrations of C–O–C.^45^ The peaks at 720 cm^−1^ corresponded to the inner rocking vibration of the –CH_2_– groups.^46^

Fig. 11(c) shows the Raman spectra of the EVA resin and solvent-free conductive Ag/EVA composite. From the Raman spectrum of the EVA resin, we can see that the peaks of aliphatic carbon–hydrogen (C–H) stretching vibrations were well resolved in the region of 2840–2940 cm^−1^.^47^ The presence of carbonyl groups was verified by the C=O asymmetric stretching vibration at 1732 cm^−1^.^48^ The typical bands at 1063 and 1128 cm^−1^ were ascribed to the C–C stretching vibration.^49^ The peaks at 1295 and 1440 cm^−1^ were ascribed to the bending and twisting modes of the ethylene groups, respectively.^50^ The feature at 634 cm^−1^ were attributed to the O–C=O deformation vibration.^51^ In the Raman spectrum of the composite, a newly observed peak at 234 cm^−1^ represented the Ag–O bond,^52^ which was generated through the oxygen atoms of the carbonyl groups coordinating with the silver atoms on the surfaces of the silver flakes. The peak at 1380 cm^−1^ signified the symmetric deformation of the methyl groups. The short distances between the surfaces of the silver flakes and the molecular chain of the ethylene vinyl acetate copolymer might have led to changes in the Raman activity of the conductive Ag/EVA composites, eventually causing the peaks at 1295 and 1440 cm^−1^ to disappear.

Fig. 11(d) illustrates the TGA results of the EVA resin and solvent-free electrically conductive Ag/EVA composite (55 wt% Ag). A two-step degradation process can be observed in the TGA curves for the EVA resin and solvent-free electrically conductive Ag/EVA composite (55 wt% Ag). The first step occurred from 240 °C to 370 °C. This step can be assigned to the loss of the acetate groups in EVA and the formation of carbon–carbon double bonds.^53,54^ The second degradation step, in the range of 410–450 °C, was ascribed to the unsaturated chain scission of the polyethylene main chains.^55^ After 500 °C, there was a slight residue in the EVA sample.

## 4. Conclusions

In summary, a solvent-free electrically conductive Ag/EVA composite was prepared for which conductive silver flake powders were prepared using the nanofilm transition method. Nanometer-thick silver flake powders with smooth and flat surfaces greatly improved the formation of the conductive network, thereby improving the electrical conductivity of the conductive Ag/EVA composites. Furthermore, the electrically conductive Ag/EVA composites could be absorbed into the pores of the paper substrate to facilitate strong binding between the two. Finally, Ag–O bonds that formed between the silver flake powders and the EVA resin are beneficial to the conduction of electrons through the conductive system. The printed conductive film together with the paper substrate has excellent flexibility. When the fill rate of the as-prepared silver flake powders reached 55 wt%, the volume resistivity of the electrically conductive composite reached 1.23 × 10^−4^ Ω cm. Because of their environmental friendliness, high electrical conductivity, excellent bending performance and adhesion, solvent-free electrically conductive Ag/EVA composites filled with silver flake powders have great potential for application in flexible and printable electronics.

## Conflicts of interest

There are no conflicts to declare.

## Supplementary Material

RA-009-C9RA02593F-s001
