# Supplementary material for: Solvent-free electrically conductive Ag/ethylene vinyl acetate (EVA) composites for paper-based printable electronics
Source: RSC Adv. 2019 Jun 21;9(34):19501–7. doi: 10.1039/c9ra02593f (PMC9065322; doi:10.1039/c9ra02593f)
Supplement: RA-009-C9RA02593F-s001 [file RA-009-C9RA02593F-s001.pdf]

Electronic Supplementary Material (ESI) for RSC Advances.
This journal is © The Royal Society of Chemistry 2019

# Supporting information

# Solvent-free electrically conductive Ag/ethylene vinyl acetate (EVA) composites for paper-based printable electronics

Yuqiu Shen,[1] Zhenxing Chen,[1,2]* Yong Zhou,[1] Zuomin Lei,[1] Yi Liu,[1] Wenchao Feng,[1] Zhuo Zhang,[1] and Houfu Chen[1]

*1. School of Chemical Engineering and Technology, Sun Yat-sen University, Tangjiawan, Zhuhai, 519082, P. R. China*

*2. The Key Laboratory of Low-Carbon Chemistry & Energy Conservation of Guangdong Province, Sun Yat-sen University, Guangzhou, 510275, P. R. China*

**Corresponding author: E-mail: 2764927916@qq.com, 3362104303@qq.com*

# Experimental Section

## Weight percent selection of silver powders of solvent-free electrically conductive Ag/EVA composites

The reason why the loading of silver flakes was fixed between 40-70 wt% is as follow: Once the silver loading of Ag/EVA composites was 35 wt%, the volume resistivity of as-prepared, FAgL 6500 and FAgL6501 reached 0.82 Ω·cm, 16.8 Ω·cm and 36.9·Ω·cm, respectively. The volume resistivity increases by three orders of magnitude when compare with the Ag/EVA composites with the loading of 40 wt%. When the loading of silver surpass 70 wt%, electrical conductivity continues to deteriorate; furthermore, the production costs of them have been greatly raised; thirdly, the stirring of Ag/EVA composites becomes much more difficult; finally, the mechanical property of cured film of Ag/EVA composites deteriorate. Considering the electrical conductivity, mechanical property and production cost, the Ag/EVA composites filling with 40-70 wt% silver powders are chosen as the research subject. The electrical percolation of the three Ag/EVA composites are different, The electrical percolation of Ag/EVA composites filling with as-prepared flaky silver powders is around 25 wt%, of FAgl6500 and FAgL 6501 is about 30 wt%.

**Table S1** The conversion relationship between weight fraction and volume fraction of solvent-free electrically conductive Ag/ethylene vinyl acetate (EVA) composites filling with different silver powders.

| Name | Tap density (g/cm$^3$) | Weight percent (wt %) | Volume fraction (vol%) |
|---|---|---|---|
| | | 40.0 | 13.4 |
| | | 45.0 | 16.0 |
| | | 50.0 | 18.9 |
| As-prepared | 4.3 | 55.0 | 22.1 |
| | | 60.0 | 25.9 |
| | | 65.0 | 30.2 |
| | | 70.0 | 35.2 |
| | | 40.0 | 18.2 |
| | | 45.0 | 21.4 |
| | | 50.0 | 25.0 |
| FAgL 6500 | 3.0 | 55.0 | 28.9 |
| | | 60.0 | 33.3 |
| | | 65.0 | 38.2 |
| | | 70.0 | 43.7 |
| | | 40.0 | 12.2 |
| | | 45.0 | 14.6 |
| | | 50.0 | 17.2 |
| FAgL6501 | 4.8 | 55.0 | 20.3 |
| | | 60.0 | 23.8 |
| | | 65.0 | 27.9 |
| | | 70.0 | 32.7 |

Note: The density of EVA is 1.0 g/cm$^3$.

## Morphology of solvent-free electrically conductive Ag/EVA composites

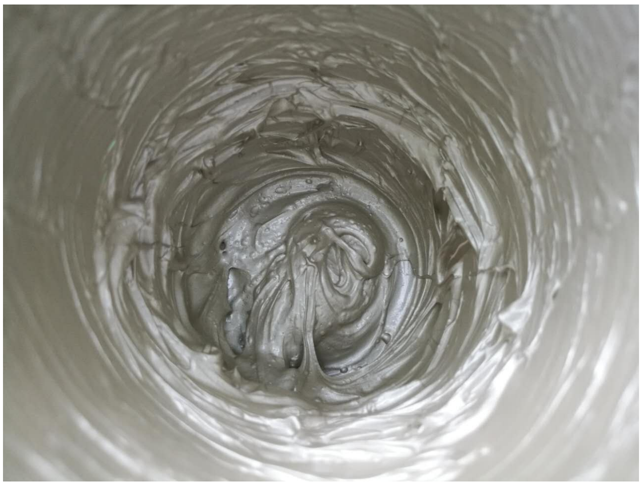

**Figure S1.** Optical images of solvent-free electrically conductive Ag/EVA composites (filled with 55wt% as-prepared flake silver powders)

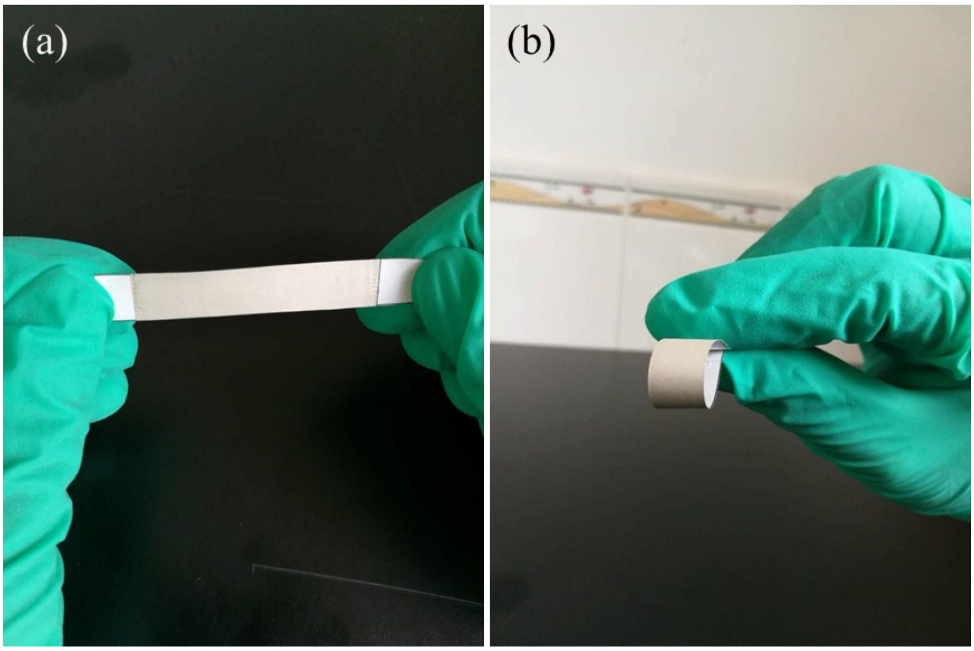


**Figure S2** Cured conductive films of solvent-free electrically conductive Ag/EVA composites (55 wt% Ag) filling with as-prepared flake silver powders.

## SEM micrographs of cured conductive films

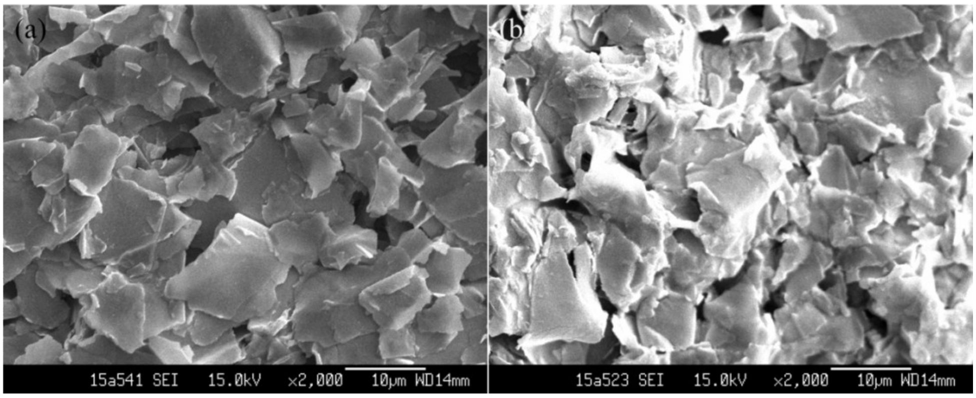


**Figure S3** SEM of solvent-free electrically conductive Ag/EVA composites filled with as-prepared flake silver powder (a) 55 wt% Ag (b) 65 wt% Ag.

## XRD analysis

X-ray diffraction was carried out to study the crystalline structures of the home-made and commercial silver powders. Figure S4 shows all the samples have four characteristic peaks for crystalline metallic silver at about 38.1°, 44.5°, 64.5°, 77.4° corresponding to the Bragg's reflection indices of (111), (200), (220), and (311) planes in a fcc structure, proving that all the silver powders haven't been oxidized.

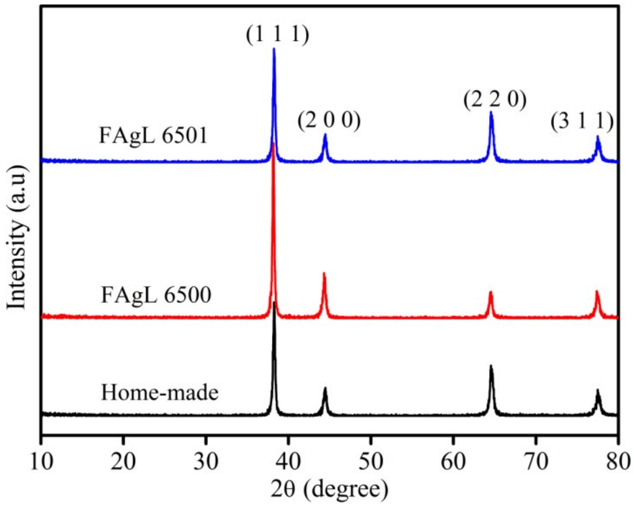


**Figure S4** X-ray diffraction (XRD) patterns of flake Ag powders.

## ATR-FTIR of paper substrate

It was observed that the peak at 880 $cm^{-1}$ was assigned to C-H bending vibrations of glycosidic linkage.[1] The peak at 1055 can be corresponded to the C-O stretch of glucose ring. The peak at 1110 and 1160 $cm^{-1}$ which could be associated to the -C-O-C- asymmetric stretch and vibration of glucose ring stretch in cellulose.[2] The Shoulder peak at 1343 and 1413 $cm^{-1}$ were assigned to the asymmetric $CH_2$ bending and wagging.[3] The band appearing at 1632 $cm^{-1}$ can be assigned to the conjugated C=O. The small peak which appeared at 1730 $cm^{-1}$ can be attributed to the -C-O- stretching of the cellulose.[4] The band at 2902 $cm^{-1}$ appeared due to C-H stretching in cellulose. The band at 3345 $cm^{-1}$ can be ascribed to the stretching of H-bonded of -OH groups.[5]

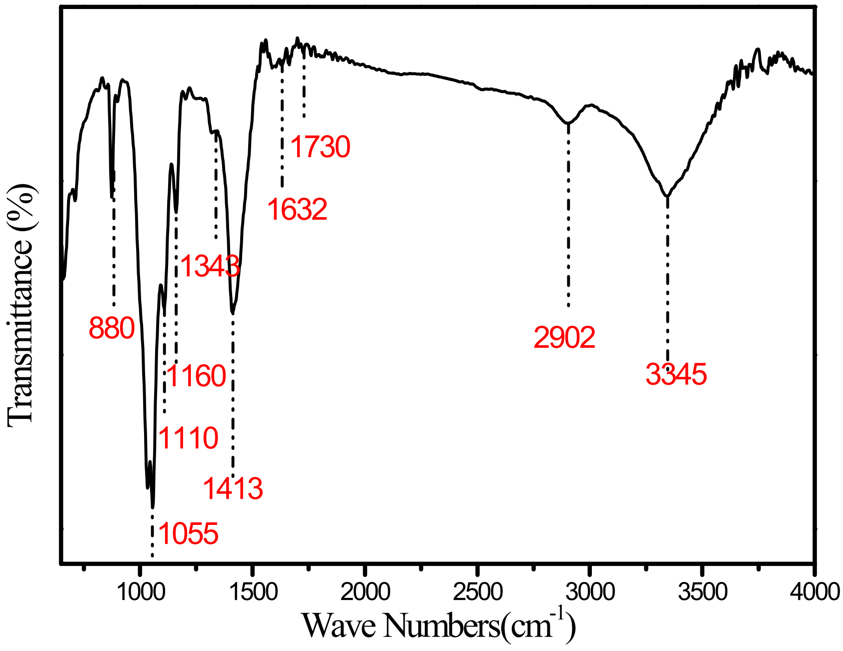


**Figure S5** ATR-FTIR spectroscopy of paper substrate.

## Adhesion test

From Figure S6(c) we can see that no noticeable conductive films were removed by the tape, a 5B level of adhesion strength was obtained. This can be ascribed to the excellent bonding of the conductive Ag/resin composites towards the paper substrate.

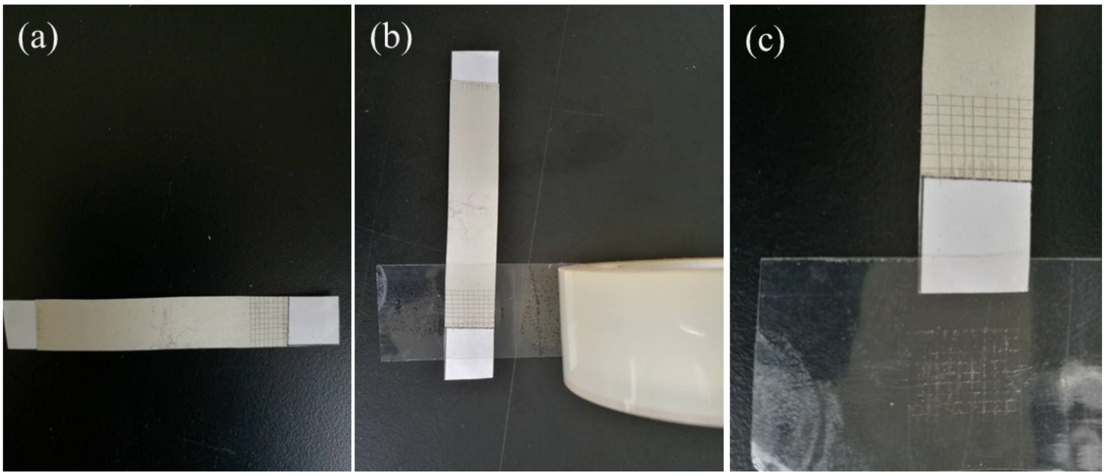


**Figure S6** Optical images of (a) cured Ag/EVA conductive film after crosshatched; (b) the adhesion of conductive Ag/EVA film was testing by Scotch tape (3M); (c) the 3M tape after removed from the conductive film.

References


1. K. Pandey, *J. Appl. Polym. Sci.*, 1999, **71**, 1969.

2. M. Kacurakova, P. Capek, V. Sasinkova, N. Wellner and A. Ebringerova, *Carbohyd. Polym.*, 2000, **43(2)**, 200.

3. D. Oldak and H. Kaczmarek, *J. Mater. Sci.*, 2005, **40**, 4192.

4. C. Cao, Z.L. Yang, L.J. Han, X.P. Jiang and G.Y. Ji, *Cellulose*, 2015, **22**, 148.

5. K. Das, D. Ray, N. R. Bandyopadhyay and S. Sengupta, *J. Polym. Environ.* 2010, **18**, 360.

6. A. Nawrocka, M. Krekora, Z. Niewiadomski and A. Miś, *Food Hydrocolloids*, 2018, **85**, 182.
